# Rekindling hopes for lithium-sulfur batteries

**DOI:** 10.1093/nsr/nwaf079

**Published:** 2025-02-28

**Authors:** Hongtao Qu, Bao-Lian Su

**Affiliations:** Laboratory of Inorganic Materials Chemistry (CMI), University of Namur, Belgium; Laboratory of Inorganic Materials Chemistry (CMI), University of Namur, Belgium; State Key Laboratory of Advanced Technology for Materials Synthesis and Processing, Wuhan University of Technology, China

Lithium-sulfur (Li-S) batteries represent a promising solution for next-generation energy storage due to their high energy density, low cost, and environmental friendliness. However, liquid electrolyte-based Li-S batteries are plagued by the ‘polysulfide shuttling’ effect, leading to severe performance degradation [1,2]. The use of solid electrolytes (SEs) to construct all-solid-state Li-S batteries (ASSLSBs) has given new hope as it not only mitigates polysulfide shuttling but also enhances the intrinsic safety of the batteries [3].

Conventional ASSLSB designs incorporate SEs and conductive carbon into the sulfur cathode to facilitate ion and electron transport. However, the sulfur redox reactions occur primarily at the three-phase boundaries of SE, active material, and conductive carbon, leaving the majority of two-phase interfaces underutilized, and the sluggish conversion kinetics between sulfur (S) and lithium sulfide (Li₂S) remains a great challenge as both materials are ionically and electronically insulating [4]. All of these significantly limit the rate performance and cycling stability (Fig. 1a). Confidence in their industrial application was thus severely lacking.

**Figure 1.**
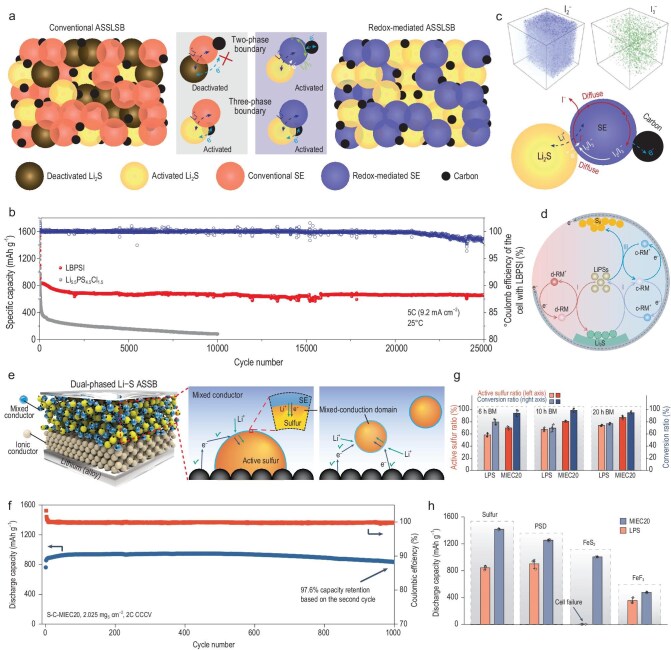
(a) Schematic showing the design principle for the fast-charging mechanism of ASSLSBs with the redox-mediated SE. (b) Long-term cycling performance of ASSLSBs based on LBPSI and Li_5.5_PS_4.5_Cl_1.5_ at 5C and 25°C. (c) Time-of-flight secondary ion mass spectrometry 3D representations of I_2_^−^ and I_3_^−^ in the fully charged sulfur cathode with LBPSI and scheme of the surficial I^−^–I_2_/I_3_^−^ redox-mediating mechanism for SSSRR. Adapted with permission from ref. [5]. (d) Schematic illustration of the working mechanism of RMs in liquid-state Li-S batteries. Adapted with permission from ref. [7]. (e) Illustration of the sulfur cathode using MIEC. (f) The cycling performance of the S-C-MIEC20 cathode at 2C and 60°C. (g) The quantified active sulfur ratio and estimated conversion ratio of active sulfur to Li_2_S in sulfur cathodes with 75Li_2_S⋅25P_2_S_5_ (LPS) or MIEC20. (h) A comparison of the discharge capacity of different conversion cathodes. Adapted with permission from ref. [6].

Two recent papers, published practically simultaneously in *Nature* and *Nature Materials*, restore hopes for the industrial application of solid-state Li-S batteries, improving the sluggish solid-solid sulfur redox kinetics by addressing confined sulfur redox reactions occurring at SE/active material/carbon three-phase boundaries through two different strategies. Pang *et al*. proposed the use of a redox-mediated SE, while Wang *et al*. introduced mixed ionic–electronic conductors (MIECs) to the sulfur cathodes [5,6].

Pang *et al*. introduced a redox-mediated glassy electrolyte, lithium thioborophosphate iodide (LBPSI), to overcome the limitations of the solid-solid sulfur redox reaction (SSSRR) at the three-phase boundaries [5]. The LBPSI electrolyte functions as both a superionic conductor and a redox mediator within the cathode composite. They demonstrated that the LBPSI electrolyte significantly improves the conversion efficiency between S and Li₂S through involving the reversible redox reactions between I⁻ and I₂/I₃⁻ during charging.

Using this redox-mediated glassy electrolyte, ultrafast SSSRR was achieved, delivering a remarkable capacity of 432 mAh g⁻¹ at 150C and 60°C. Additionally, the assembled ASSLSBs demonstrated exceptional cycling stability over 25 000 cycles with 80.2% capacity retention at room temperature (Fig. 1b). This superior performance can be ascribed to the multifunctional role of the LBPSI electrolyte. At fast charging rates, I₂ and I₃⁻ are formed on the LiBPSI electrolyte surface due to the oxidation of LiBPSI (Fig. 1c). The generated I₂ can diffuse rapidly and redistribute at adjacent Li₂S/SE two-phase boundaries, where it chemically oxidizes Li₂S to S. Thus, LiBPSI not only provides ion conduction pathways but also forms a surface I⁻–I₂/I₃⁻ redox couple, enhancing the conversion of Li₂S to S. Interestingly, the redox mediator is ineffective at low charging rates, as the required potential cannot be reached. Redox mediation is generally recognized as an effective strategy for enhancing cathode kinetics in liquid-state Li-S batteries (Fig. 1d) [7]. The introduction of redox mediators improves the conversion efficiency between S and Li₂S₂/Li₂S, thereby enhancing rate capability and sulfur utilization. In ASSLSBs, it remains debated whether S₈ undergoes a direct conversion to Li₂S without forming polysulfide intermediates, which partially results in sluggish sulfur redox kinetics compared to liquid-state systems.

While large-format ASSLSB have not been demonstrated, the innovation serves both as a Li-ion conductor and a redox mediator, transforming restricted three-phase interfacial reactions into more favorable two-phase interfacial reactions in order to improve the sluggish solid-solid sulfur redox kinetics. This innovative work allows industries to regain confidence in Li-S batteries and equally opens new horizons for the development of fast-charging ASSLSBs.

Building on a similar idea, Wang *et al.* proposed the use of MIECs in sulfur cathodes. These MIECs replace conventional SEs, enabling conversion reactions at sulfur–MIEC interfaces in addition to traditional three-phase boundaries (Fig. 1e). The addition of TiS₂ in MIECs improves electronic conductivity while facilitating sulfur redox kinetics at sulfur–MIEC boundaries, resulting in increased active sulfur ratios and conversion efficiencies (Fig. 1g). As a result, ASSLSBs incorporating MIECs with high sulfur content (50 wt.%, 2.025 mg cm^−2^) and low carbon content (10 wt.%) achieved a discharge capacity exceeding 1450 mAh g⁻¹ and over 1000 cycles with an impressive capacity retention of 97.6% (Fig. 1f). Furthermore, the authors applied MIEC20 to other high-energy, affordable conversion cathodes (Fig. 1h), achieving enhanced active material utilization and improved rate performance, highlighting the versatility of this strategy. The sulfur content and loading in this work needs further improvement to compete with state-of-the-art Li-ion batteries.

For practical applications, higher sulfur content and loading are essential. It is estimated that ASSLSBs can achieve a gravimetric energy density of ∼743 Wh kg⁻¹ with a cathode loading of 6 mg cm⁻² and a sulfur content of 80 wt%. This value can increase to as high as 1100 Wh kg⁻¹ with a loading of 18 mg cm⁻² [3]. However, in ASSLSBs, the sluggish solid-state diffusion of ions and electrons across the sulfur cathode limits sulfur utilization and fast-charging capability, especially as the cathodes become thicker [6]. Intriguingly, this work demonstrated that incorporating MIECs into solid sulfur composite cathodes can not only enhance ion and electron transport but also reactivate ‘dead’ sulfur species isolated from Li-ion and electron pathways. The proposed strategy provides an effective solution to completely solve the sluggish reaction kinetics bottleneck at the traditional three-phase boundary in solid sulfur cathodes.

In summary, both studies highlight the critical role of addressing interfacial challenges in sulfur cathode composites for ASSLSBs. The insulating nature of sulfur species necessitates the innovative engineering of three-phase boundaries to achieve durable sulfur cathodes with rapid redox kinetics. These two studies give clear guidance to develop more efficient redox-mediated electrolytes and MIECs for fast-charging and energy-dense ASSLSBs and restore confidence in Li-S batteries and help to develop other types of promising batteries [2,8].

## References

[1] Yan M, Dong W, Liu F et al. Natl Sci Rev 2022; 9: nwac078. 10.1093/nsr/nwac078 35832774 PMC9273299

[2] Song H, Li T, He T et al. Chem Synth 2023; 3: 40. 10.20517/cs.2023.22

[3] Kim JT, Su H, Zhong Y et al. Nat Chem Eng 2024; 1: 400–10. 10.1038/s44286-024-00079-5

[4] Zhou J, Holekevi Chandrappa ML, Tan S et al. Nature 2024; 627: 301–5. 10.1038/s41586-024-07101-z 38448596

[5] Song H, Münch K, Liu X et al. Nature 2025; 637: 846–53. 10.1038/s41586-024-08298-9 39814898

[6] Wang D, Gwalani B, Wierzbicki D et al. Nat Mater 2025; 24: 243–51. 10.1038/s41563-024-02057-x 39762497

[7] Pang YQ, Zhao M, Chen Z-X et al. Batter Supercaps 2022; 5: e202100359. 10.1002/batt.202100359

[8] Wang H, Yan W, Zhu M. Chem Synth 2024; 4: 57. 10.20517/cs.2024.67

